# Oral Microbiota, the Oral–Brain Axis, and Neurodegeneration: Mechanisms and Dietary Modulation

**DOI:** 10.3390/antiox15080925

**Published:** 2026-07-25

**Authors:** Justyna Godos, Giuseppe Caruso, Giuseppe Mainas, Agnieszka Micek, Andrea Di Mauro, Lucia Buccarello, Nohora Milena Martínez López, Evelyn Frias-Toral, Francesca Giampieri, Andrea Lehoczki, Gaetano Isola, Fabio Galvano, Zoltan Ungvari, José L. Quiles, Maurizio Battino, Giuseppe Grosso

**Affiliations:** 1 Department of Biomedical and Biotechnological Sciences, University of Catania, 95123 Catania, Italy; 2 Departmental Faculty of Medicine, UniCamillus-Saint Camillus International University of Health Sciences, 00131 Rome, Italy; giuseppe.caruso@unicamillus.org (G.C.);; 3 IRCCS San Camillo Hospital, 30126 Venice, Italy; 4 Periodontology Unit, Centre for Host Microbiome Interactions, Faculty of Dentistry, Oral & Craniofacial Sciences, King’s College London, London SE1 9RT, UK; 5 Statistical Laboratory, Jagiellonian University Medical College, 31-126 Kraków, Poland; 6 Research Group on Food, Nutritional Biochemistry and Health, Universidad Europea del Atlántico, Isabel Torres 21, 39011 Santander, Spain; 7 Universidad Internacional Iberoamericana, Campeche 24560, Mexico; 8 Universidad Internacional Iberoamericana, Arecibo, PR 00613, USA; 9 Fundación Universitaria Internacional de Colombia, Bogotá 111321, Colombia; 10 Escuela de Medicina, Universidad Espíritu Santo, Samborondón 0901952, Ecuador; 11 Dipartimento di Scienze Cliniche Specialistiche e Odontostomatologiche, Università Politecnica delle Marche, Via Ranieri, 60131 Ancona, Italy; 12 Joint Laboratory on Food Science, Nutrition, and Intelligent Processing of Foods, Polytechnic University of Marche, Italy, Universidad Europea del Atlántico Spain and Jiangsu University, China at the Polytechnic University of Marche, 60131 Ancona, Italy; 13 International Training Program in Geroscience, Doctoral College/Institute of Preventive Medicine and Public Health, Semmelweis University, 1085 Budapest, Hungary; 14 Fodor Center for Prevention and Healthy Aging, Semmelweis University, 1085 Budapest, Hungary; 15 Institute for Translational Research, 1085 Budapest, Hungary; 16 Unit of Periodontology, Department of General Surgery and Surgical-Medical Specialties, School of Dentistry, University of Catania, 95123 Catania, Italy; gaetano.isola@unict.it; 17 Vascular Cognitive Impairment, Neurodegeneration and Healthy Brain Aging Program, Department of Neurosurgery, University of Oklahoma Health Sciences Center, Oklahoma City, OK 73104, USA; 18 Department of Physiology, Institute of Nutrition and Food Technology “José Mataix Verdú”, Biomedical Research Centre, University of Granada, 18100 Armilla, Spain

**Keywords:** oral microbiota, dysbiosis, periodontitis, neuroinflammation, cognition, brain, diet, polyphenols

## Abstract

The oral microbiota represents a complex and dynamic microbial ecosystem that plays a critical role in preserving both oral and systemic homeostasis. Emerging evidence suggests that alterations in oral microbial milieu (dysbiosis) may contribute to the pathogenesis of neurodegenerative disorders, especially Alzheimer’s disease (AD), through the oral–brain axis. This review synthesizes current evidence on the pathways linking oral microbiota to cognitive decline, integrating microbial, immunological, and vascular perspectives. Oral pathogens may access the central nervous system via hematogenous dissemination or neural routes, including the trigeminal nerve, while simultaneously promoting systemic inflammation, immune activation, and blood–brain barrier disruption. These processes converge on key neurodegenerative mechanisms, including chronic neuroinflammation, amyloid-β accumulation, and tau pathology. In parallel, alterations in oral microbial composition have been linked to disease severity, supporting a potential role of dysbiosis in both initiation and progression of cognitive impairment. Diet emerges as a critical modifiable determinant of oral microbial ecology. Diets rich in refined sugars may promote dysbiosis and inflammatory signaling, whereas (poly)phenols, probiotics, and prebiotics may support microbial eubiosis and exert neuroprotective effects through modulation of host–microbe interactions. Although current evidence remains largely observational and mechanistic, the diet–oral microbiota–brain axis represents a promising target for preventive and therapeutic strategies aimed at mitigating cognitive decline and promoting healthy aging. Future longitudinal and interventional studies are required to establish causality and translate these insights into clinical practice.

## 1. Introduction

Aging-related cognitive decline and neurodegenerative disorders, particularly Alzheimer’s disease (AD), represent a major and growing global health challenge [1]. While traditionally viewed through the lens of neuronal pathology, centered on amyloid-β accumulation and tau hyperphosphorylation, AD is increasingly recognized as a multifactorial disorder shaped by systemic processes, including chronic inflammation, microvascular dysfunction, and host–microbiome interactions [2]. Within this emerging framework, peripheral biological systems are no longer considered passive bystanders, but active contributors to brain aging and neurodegeneration [3].

The human oral cavity is home to one of the most complex and diverse microbial ecosystems in the body [4]. Known collectively as the oral microbiota, this community comprises hundreds of bacterial species along with fungi, archaea, viruses, and protozoa, residing in distinct ecological niches including the teeth, gingiva, tongue dorsum, cheeks, hard and soft palate, and saliva [4]. Under physiological conditions, the oral microbiota contributes to immune homeostasis, metabolic function, and colonization resistance. However, disruption of this balanced ecosystem (dysbiosis), most commonly observed in periodontal disease, can trigger local and systemic inflammatory responses with far-reaching consequences. Increasing evidence suggests that oral microbial dysbiosis may extend beyond the oral cavity and influence distant organs, including the vasculature and the central nervous system (CNS) [5]. Periodontal pathogens, particularly those associated with chronic periodontitis (e.g., *Porphyromonas gingivalis*, *Fusobacterium nucleatum*, *and Treponema denticola*), have been implicated in systemic inflammatory responses and chronic diseases, including cardiovascular disease, diabetes, rheumatoid arthritis, and more recently, neurodegenerative disorders [6].

A growing body of experimental and clinical evidence supports the existence of an oral–brain axis, whereby oral pathogens and their virulence factors contribute to neurodegenerative processes through multiple, interconnected mechanisms [7]. These include hematogenous dissemination of bacteria and endotoxins, neural transmission via cranial nerves such as the trigeminal pathway, and the induction of systemic inflammation that compromises blood–brain barrier integrity. Once in the brain, these processes can promote microglial activation, chronic neuroinflammation, and the accumulation of hallmark AD pathologies, including amyloid-β plaques and tau aggregates [8,9,10]. Notably, key periodontal pathogens such as *Porphyromonas gingivalis* have been detected in the postmortem brains of patients with AD, while their proteolytic enzymes (gingipains) have been linked to neurodegenerative processes in preclinical models [11]; nevertheless, these findings do not establish a causal relationship between the presence of these microorganisms in the brain tissue and the development or progression of the disease.

Furthermore, dietary habits significantly modulate the composition and function of the oral microbiota, thereby influencing both oral and brain health [12]. Diets high in refined sugars and saturated fats promote the proliferation of pathogenic microbes and inflammatory responses [13], whereas diets rich in fiber, polyphenols, and unsaturated fats, such as the Mediterranean diet, support an eubiotic (balanced) microbiota and may exert neuroprotective effects [13,14]. Fiber and polyphenols may play an important role as prebiotic-like agents, promoting microbial metabolic transformations that generate bioactive metabolites potentially involved in cognitive health [15]. However, while their bidirectional relationship with the gut microbiota has been extensively investigated, the interplay with the oral microbiota remains comparatively underexplored [16].

Taken together, these observations highlight the emerging concept of the oral microbiota–diet–brain axis as a promising framework in neurodegeneration research. Given these converging lines of evidence, it is critical to explore how alterations in the oral microbiome may influence the onset and progression of AD and to assess how dietary interventions could modulate this relationship [17]. This review aims to provide a comprehensive overview of the existing evidence linking the oral microbiota to brain health, with a focus on dementia and Alzheimer’s disease–associated outcomes.

## 2. The Oral Microbiota: Composition and Function

Among the bacterial taxa inhabiting the oral cavity, Streptococcus is the most abundant and functionally significant genus [18]. Early colonizers such as *Streptococcus gordonii*, *S. oralis*, *S. sanguinis*, *S. mutans*, and *S. sobrinus* play a central role in initiating dental biofilm formation [19]. These bacteria adhere to the salivary-acquired pellicle (a glycoprotein-rich film coating tooth surfaces) through a combination of molecular forces, including hydrogen bonding, electrostatic interactions, and hydrophobic effects [20]. This initial colonization establishes a microenvironment conducive to the adhesion and growth of secondary colonizers, including species from Granulicatella, Gemella and Rothia [21]. These polymicrobial interactions give rise to a mature biofilm characterized by complex cooperative and antagonistic relationships, quorum sensing, metabolic synergy, and spatial structuring [22].

The oral microbiota constitutes a critical component of the human microbiome, performing a variety of physiological and immunological functions that extend beyond the oral cavity [23]. Under homeostatic conditions, this microbial community provides essential protective roles through mechanisms of colonization resistance, whereby commensal organisms inhibit the adhesion, proliferation, and invasion of pathogenic and opportunistic microbes [24]. This protective barrier function prevents microbial translocation, the penetration of microbial toxins, and the systemic dissemination of xenobiotics, thereby contributing to both oral and systemic immune defense [25]. Beyond this defensive role, the oral microbiota engages in a wide range of metabolic, structural, and immunomodulatory activities [25]. These include the regulation of gas composition within host cavities, morphokinetic influences on epithelial tissues, and the production of a suite of enzymes involved in the metabolism of proteins, carbohydrates, lipids, and nucleic acids [26,27]. Commensal microbes are also known to synthesize biologically active compounds such as vitamins, short-chain fatty acids, antimicrobial peptides, and other metabolites that can affect host physiology and immunity [28]. Additionally, the oral microbiome serves as a reservoir for microbial genetic material, including plasmids and chromosomal genes, which may influence horizontal gene transfer and microbial adaptability [29].

Saliva plays a central role in maintaining the ecological stability of the oral microbiota, serving as a continuous source of nutrients including amino acids, proteins, glycoproteins, peptides, and vitamins [30]. Alongside saliva, gingival crevicular fluid (GCF), an exudate derived from periodontal tissues, further supports microbial growth by delivering host-derived nutrients that sustain microbial activity, particularly in subgingival environments [31]. These fluids form the primary nutrient sources for oral microbes, especially given the transient and intermittent nature of dietary contact with the oral cavity. While mastication can stimulate salivary flow and modulate oral pH, the nutritional needs of resident microbes are largely met by endogenous sources rather than food itself [31]. Streptococci, particularly early colonizers such as *Streptococcus gordonii* and *S. oralis*, are highly adapted to utilize glycoproteins in saliva as sources of sugar residues, thereby playing a key role in nutrient acquisition and biofilm development [32]. Although the evidence that diet may exert an immediate direct effect on the short-term proliferation of oral bacteria is limited, this does not preclude its longer-term influence on the oral microbial ecosystem [13]. Rather than acting through acute changes in bacterial growth, repeated and sustained dietary exposures may exert cumulative ecological pressure by altering nutrient and substrate availability, salivary composition, local pH, host immune function, and inflammatory responses. Over time, these indirect mechanisms can shape the composition, metabolic activity, and ecological balance of the oral microbiota, thereby contributing to microbial adaptation and oral health outcomes [13]. As the oral microbiota plays an essential role in maintaining oral and systemic homeostasis, preserving its ecological balance is critical, as it is susceptible to disruption. Alterations in its composition can lead to dysbiosis with local and systemic consequences. Emerging evidence suggests that these alterations may extend beyond the oral cavity, potentially impacting neurological and brain health.

## 3. Oral Microbiota Dysbiosis and Brain Health

The oral microbiota, under healthy conditions, functions as a dynamic yet balanced community that contributes to immune modulation, nutrient metabolism, and pathogen constraint [33]. A key component of this defensive capacity is colonization resistance [34], a phenomenon essential for maintaining microbial homeostasis in the oral cavity. It refers to the ecological stability and specificity provided by the resident microbiota, which act to prevent the establishment and proliferation of exogenous or pathogenic species [35]. When colonization resistance is compromised due to factors, such as poor oral hygiene, systemic disease, immunosuppression, or antibiotic use, there is a notable shift in microbial composition [35]. This dysbiosis is often characterized by a reduction in beneficial “core” commensal taxa and a concurrent rise in potentially pathogenic organisms [36]. Such microbial imbalances can facilitate the translocation of pathogens into deeper tissues and systemic circulation, potentially triggering localized or systemic inflammatory responses [37].

Among the most common manifestations of oral dysbiosis are dental caries and periodontal disease (PD), both of which are associated with microbial imbalance and chronic inflammation [38]. Beyond their local effects, these conditions may have broader systemic implications, as poor oral health has been linked to systemic inflammation and cognitive impairment, especially in older adults [39]. Periodontitis, in particular, is a progressive, multifactorial inflammatory condition characterized by the destruction of the periodontal ligament and alveolar bone [40]. It is driven by a microbial shift from commensal to pathogenic communities, notably Gram-negative anaerobes such as *Porphyromonas gingivalis*, *Tannerella forsythia*, and *Aggregatibacter actinomycetemcomitans* [41]. These pathogens express virulence factors that facilitate tissue invasion, immune evasion, and the induction of pro-inflammatory cytokines such as interleukin-1α (IL-1α), IL-1β, IL-6, tumor necrosis factor-α (TNF-α), and prostaglandins [42]. The inflammatory milieu is further sustained by matrix metalloproteinases (MMPs), which degrade extracellular matrix components, leading to the clinical hallmarks of PD: gingival recession, periodontal pocket formation, and ultimately, tooth loss [42].

Periodontal pathogens and their inflammatory products do not remain confined to the oral cavity. Increasing evidence suggests that they can enter the systemic circulation and reach distant organs, including the brain [43,44]. Bacteremia may be induced by routine activities such as tooth brushing, flossing, or dental procedures, especially in individuals with compromised oral barriers [45]. Once in the bloodstream, these microbes or their components (e.g., lipopolysaccharides, gingipains) may traverse the blood–brain barrier (BBB) or exploit alternative neural routes to access the CNS [46]. In the first scenario, inflammatory cytokines and bacterial endotoxins released from periodontal sites can increase BBB permeability by disrupting endothelial tight junctions and activating endothelial and glial cells [46]. Unhealthy diets and age-related changes in BBB integrity, such as diminished tight junction expression, altered transporter function, and increased oxidative stress, an imbalance between excess free radicals and insufficient antioxidants in the body [47], further exacerbate this vulnerability in elderly individuals [46]. In addition to direct bloodstream access, oral microbes can exploit neural pathways. The trigeminal nerve, which provides sensory innervation to the oral and maxillofacial region and connects directly to the brainstem, represents a conduit through which pathogens may ascend to the CNS [48]. Within the trigeminal ganglia, glial cells recognize and process bacterial antigens, secrete cytokines, and potentially facilitate pathogen transport along axons via vesicular trafficking [49]. Certain intracellular pathogens are capable of evading phagocytic destruction and persisting within host cells, facilitating their migration to neural tissues [43].

**Figure 1:**
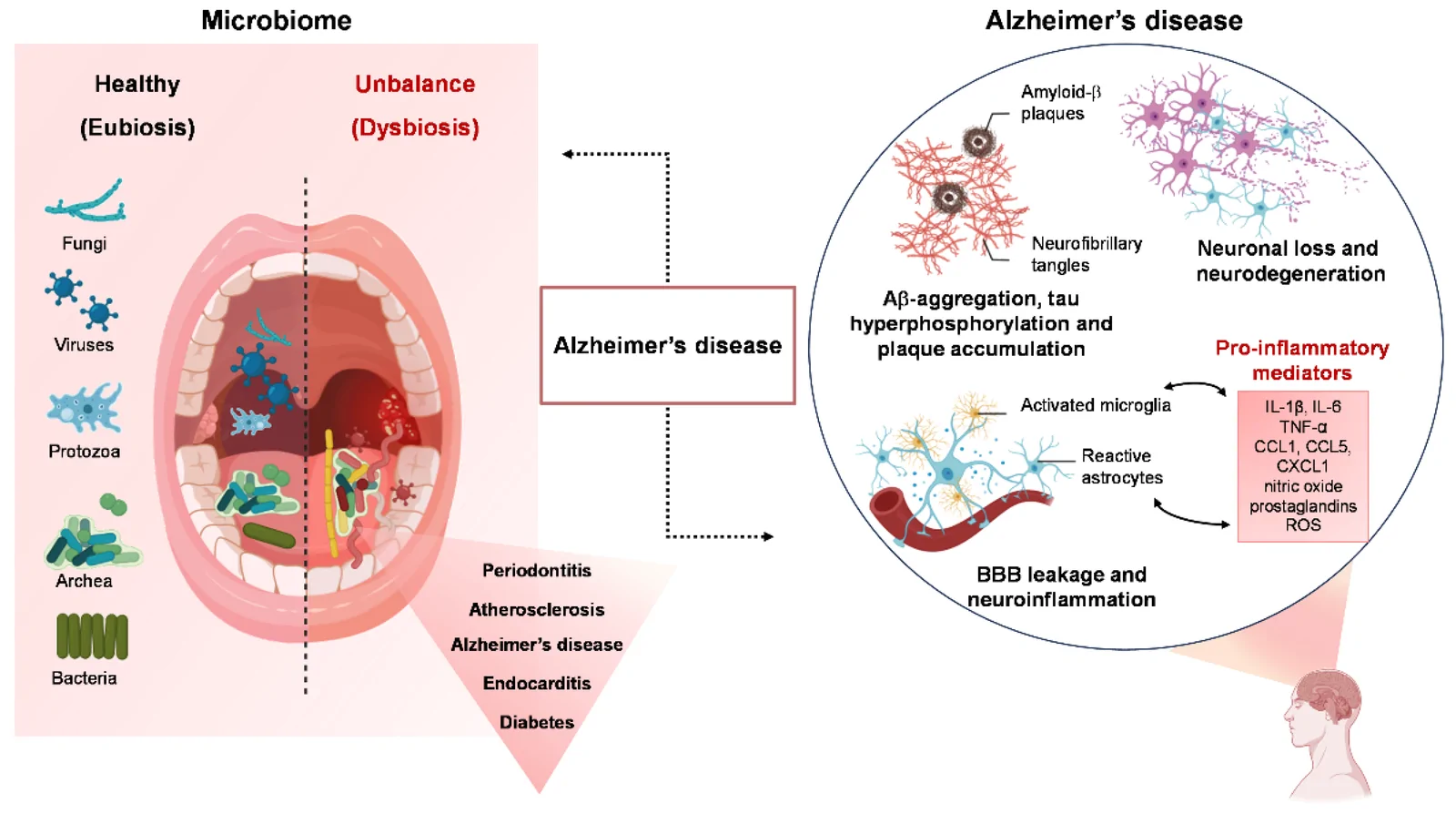
Role of oral microbiota and its involvement in Alzheimer’s disease. Schematic overview of the interplay between oral microbiota and the development of Alzheimer’s disease (AD). In healthy conditions (eubiosis), the oral cavity hosts a diverse microbial species, including bacteria, archaea, fungi, viruses, and protozoa, that maintains microbial homeostasis. Dysbiosis, commonly associated with periodontal disease, may contribute to systemic disorders such as diabetes, atherosclerosis, endocarditis, and neurodegenerative diseases. Oral microbial imbalance can induce systemic inflammation and increased blood–brain barrier permeability, enabling neuroinflammatory processes. In the brain, activation of microglia and astrocytes triggers the release of pro-inflammatory mediators (e.g., IL-1β, IL-6, TNF-α, chemokines, nitric oxide, prostaglandins, and reactive oxygen species), furthering to amyloid-β accumulation, tau hyperphosphorylation, neurofibrillary tangle formation, and progressive neuronal loss hallmarks of AD.

The entry of oral pathogens and their inflammatory byproducts into the CNS is thought to initiate or exacerbate neuroinflammation, a process central to the pathogenesis of AD [50]. Neuroinflammation is characterized by the activation of microglia and astrocytes, the release of pro-inflammatory mediators such as IL-1β, IL-6, TNF-α, chemokines (e.g., CCL5, CXCL10), nitric oxide (NO), prostaglandins, and reactive oxygen species (ROS) [51]. While neuroinflammation may initially serve a protective function, chronic activation of glial cells contributes to synaptic loss, neuronal dysfunction, and the accumulation of neuropathological hallmarks of AD, including β-amyloid (Aβ) plaques and hyperphosphorylated tau tangles (Figure 1) [51]. The aberrant aggregation of Aβ as well as that of other amyloid peptides has been linked to numerous disorders [52]. Recent studies have revealed that oral pathogens may directly influence amyloidogenesis [53]. In line with this, *P. gingivalis* and its gingipains have been detected in postmortem brain tissue of AD patients and have been shown to promote Aβ aggregation and tau pathology in murine models [54]. In parallel, the Aβ peptide itself-long implicated in AD pathophysiology-has been proposed to function as an antimicrobial peptide within the innate immune system, suggesting that its overproduction in AD may be an immune response to chronic microbial insult [55].

Microbial dysbiosis in the oral cavity appears to be correlated to AD severity. Comparative microbiome analyses have shown that individuals with AD exhibit significant alterations in oral microbial composition compared to cognitively healthy controls [56]. Specifically, increased abundances of Firmicutes (families *Veillonellaceae* and *Streptococcaceae*) and Fusobacteria (*Leptotrichiaceae*) have been reported in patients with mild to moderate AD [57]. In contrast, taxa belonging to Proteobacteria are reduced in AD, particularly in its more advanced stages [57], which suggests that disease progression may be accompanied by a shift toward a pro-inflammatory oral microbiota. In addition to taxonomic changes, oral microbial diversity is reduced in individuals with AD, reflecting ecological instability and potential functional deficits [56]. Studies have also noted that AD patients tend to have greater dental plaque accumulation, higher numbers of missing teeth, and worse overall oral hygiene, factors that contribute to microbial dysbiosis and inflammation [58]. Notably, elevated serum antibody titers against periodontal pathogens have been detected in AD patients, supporting the notion of systemic microbial exposure and immune activation [59]. Experimental studies provide further support for a potential causal role of oral microbiota in AD pathogenesis [60]. In murine models, oral administration of *P. gingivalis* or gingipain has been shown to induce classical AD-like features, including neuroinflammation, microgliosis, astrogliosis, and Aβ plaque formation [61,62,63]. Similarly, transgenic AD mice exposed to salivary microbiota collected from patients with periodontitis exhibited accelerated cognitive decline, increased brain Aβ accumulation, and elevated markers of neuroinflammation [64]. These findings suggest that oral dysbiosis may not only be a consequence of neurodegenerative disease but also a contributing factor in its initiation and progression, with increasing attention directed toward modifiable factors such as diet [65].

## 4. Oral Microbiota and Brain Function: The Potential Role of Diet

Diet plays a pivotal role in shaping the composition, structure, and function of the oral microbiota. As human dietary patterns have evolved from hunter–gatherer diets rich in fibrous plant material and low in fermentable carbohydrates to modern diets high in refined sugars and processed foods, substantial shifts in oral microbial communities have been observed [66]. These changes reflect not only the nutritional substrates available to oral microorganisms but also the selective pressures imposed by diet, favoring the growth of taxa that are best adapted to exploit specific nutrients or to thrive under conditions created by frequent sugar exposure [66]. The oral microbiota derives its primary nutritional support not from dietary intake *per se* (since food remains in the oral cavity only briefly) but from host-derived fluids, such as saliva and GCF [67]. However, frequent consumption of sugar-rich foods and beverages disrupts this equilibrium. Simple sugars serve as substrates for acidogenic and aciduric microorganisms such as Streptococcus mutans, leading to acid production, enamel demineralization, and subsequent development of dental caries [68]. Additionally, diets high in polyphenol-rich foods, such as tea, wine, coffee, and cranberries, may exert antimicrobial effects that could counteract pathogenic microbial activity and suppress virulence factors [69].

Growing interest in the therapeutic modulation of the oral microbiota has led to the exploration of dietary components, including prebiotics and phytochemicals, as well as probiotics, as potential strategies to prevent oral diseases and influence systemic conditions [70,71], including neurodegenerative disorders such as AD [12]. The rationale behind these interventions lies in their capacity to restore or maintain microbial eubiosis and reduce systemic inflammation, a known contributor to AD pathophysiology [72]. However, current evidence is derived predominantly from preclinical studies, with relatively few clinical investigations available to investigate their efficacy and translational potential in humans.

**Figure 2:**
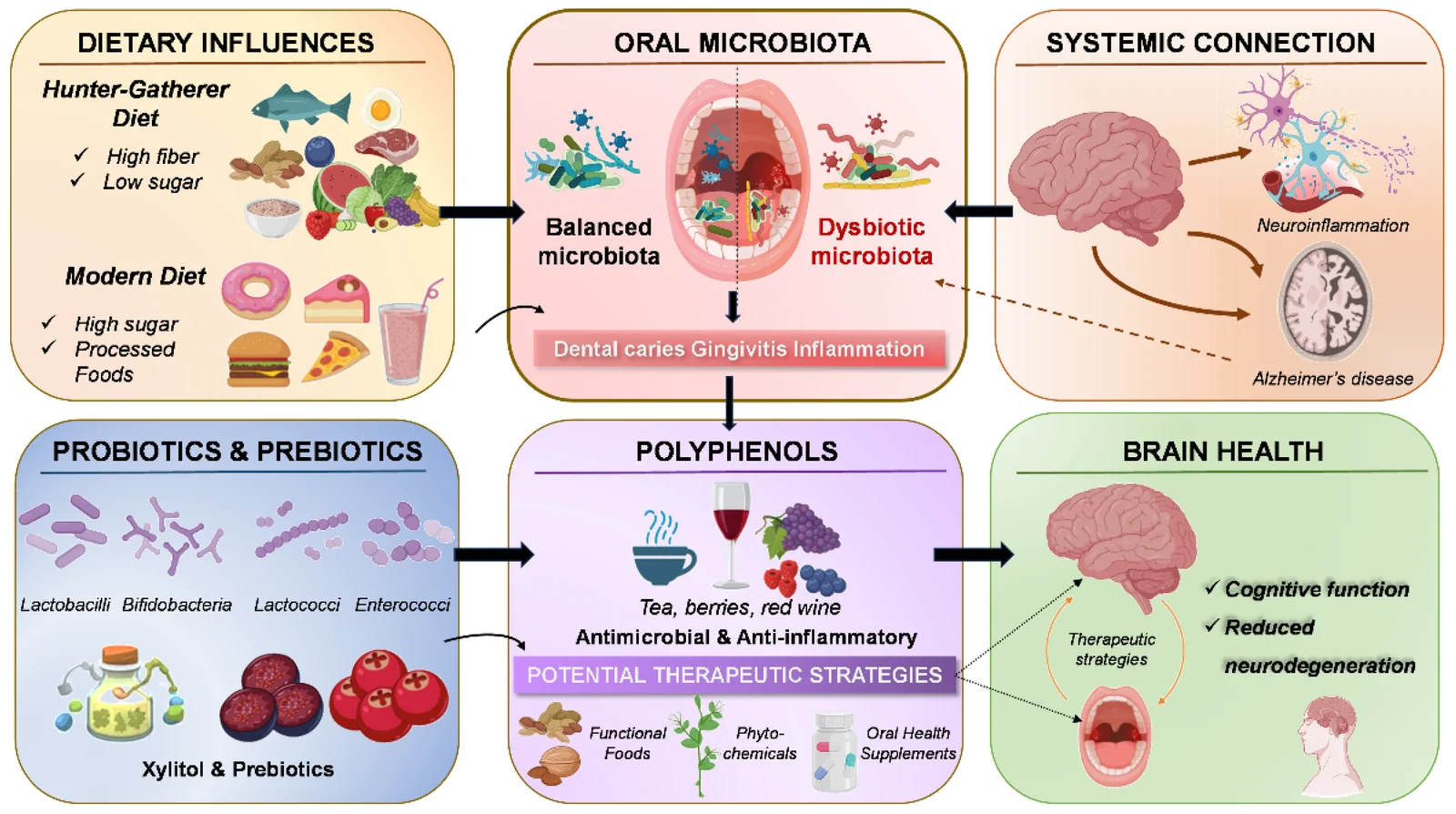
Role of dietary factors on oral microbiota modulation and potential strategies for brain health. Crosstalk among diet, oral microbiota, and brain health highlighting potential strategies to tackle neurodegenerative diseases. Dietary factors shape oral microbial balance: fiber-rich, low-sugar diets promote microbial homeostasis, whereas modern diets high in sugar and processed foods cause dysbiosis and oral inflammatory conditions such as dental caries and gingivitis. Oral dysbiosis may contribute to systemic inflammation and neuroinflammatory pathways associated with AD. Strategies to restore microbial balance include probiotics and prebiotics (e.g., *Lactobacilli*, *Bifidobacteria*, *Lactococci*, *Enterococci*, and xylitol) and polyphenol-rich foods (e.g., tea, berries, and red wine), which may support oral microbial homeostasis and cognitive function while reducing neurodegeneration.

Prebiotics, nondigestible food components that selectively stimulate the growth or activity of beneficial bacteria have been proposed as adjuncts to probiotics in managing oral and systemic health [73]. While extensively studied in the gut [74], oral-specific prebiotic research is limited. Traditional prebiotics such as inulin, fructo-oligosaccharides (FOS), and galacto-oligosaccharides (GOS) are effective in the gut [74,75], but their suitability and efficacy in the oral cavity remain less clear [76]. Nonetheless, compounds like arabinose, xylose, and xylitol have shown promise as oral prebiotics in in vitro models by inhibiting the growth of *C. albicans* and *P. gingivalis* [77]. The identification of prebiotics that selectively promote health-associated oral bacteria such as *Lactobacillus gasseri*, *L. salivarius*, *L. fermentum*, and various *Bifidobacterium* species may offer new avenues for periodontal health promotion [23]. Among dietary components, polyphenols have garnered significant attention for their antimicrobial, anti-inflammatory, and antioxidant properties [78]. These bioactive compounds, present in tea, berries, red wine, and certain herbs [79], can inhibit the growth and virulence of cariogenic and periodontopathogenic microorganisms. For example, catechins in green and black tea have been shown to suppress biofilm formation and cell viability of *S. mutans* and *S. sobrinus* [80,81]. Cranberry proanthocyanidins have been shown to inhibit glucosyltransferase activity, thereby reducing extracellular polysaccharide (EPS) synthesis, which is essential for biofilm stability and cariogenic potential [82]. Polyphenols, including chlorogenic acid, theaflavins, and baicalein, have demonstrated antibacterial activity against periodontal pathogens including *P. gingivalis*, *A. actinomycetemcomitans*, *F. nucleatum*, *S. mitis*, and *Staphylococcus aureus* [78]. Their effects include inhibition of bacterial adhesion, interference with metabolic enzymes, and suppression of inflammatory mediators [78]. Moreover, polyphenols may modulate the structure and function of EPS matrices [78]. In addition to local oral effects, polyphenols and other functional dietary components may have systemic implications. By reducing oral inflammation and pathogen load, these compounds may decrease the risk of bacteremia and systemic immune activation [78], both of which have been implicated in the pathogenesis of AD (Figure 2) [83]. In parallel, probiotics, defined as live microorganisms that confer health benefits to the host when administered in adequate amounts, have shown promise in oral health applications [84]. In the oral cavity, probiotics must adhere to non-shedding surfaces such as teeth, integrate into existing biofilms [85], and ideally avoid sugar fermentation to prevent caries development. Mechanistically, they act through competitive exclusion of pathogens, secretion of bacteriocins and other inhibitory substances, modulation of immune responses, and enhancement of epithelial barrier function [86]. Specific probiotic strains including *S. salivarius*, *Lactobacillus rhamnosus*, *L. reuteri*, *L. paracasei*, *L. casei*, *Bifidobacterium animalis*, and *B. bifidum* have been evaluated for their efficacy in reducing cariogenic bacterial load and preventing caries [87]. For instance, it has been demonstrated that supplementation with *L. rhamnosus GG* in children led to a reduction in S. mutans counts and fewer caries lesions [88]. However, other studies have reported inconsistent results [89]. Factors such as the strain used, treatment duration, administration method (monostrain vs. multistrain), follow-up time, and the endpoints measured (e.g., microbial counts vs. clinical outcomes) contribute to this variability [89,90]. Notably, an observed reduction in *S. mutans* levels does not necessarily correlate with reduced caries incidence, highlighting the complexity of evaluating oral probiotic efficacy [91].

Furthermore, certain probiotics and dietary interventions have shown direct cognitive benefits; however, current evidence so far does not support the conclusion that these effects are mediated through direct modulation of the oral microbiota. A randomized clinical trial involving older adults with AD reported that 12 weeks of daily probiotic supplementation (including *L. acidophilus*, *L. casei*, *L. fermentum* and *B. bifidum*) significantly improved Mini-Mental State Examination (MMSE) scores, reduced serum markers of oxidative stress (malondialdehyde), and decreased inflammatory biomarkers (high-sensitivity C-reactive protein) [92]. A subsequent randomized, double-blind trial in AD found that co-supplementation with a multi-strain probiotic and selenium for 12 weeks similarly improved MMSE scores and favorably modified metabolic and genetic markers [93]. Beyond Alzheimer’s dementia, several studies in older adults with memory complaints or mild cognitive impairment (MCI) have shown that supplementation with Bifidobacterium breve strains can enhance specific cognitive domains and, in some cases, slow neurodegenerative changes. B. breve A1 improved memory-related test performance in older adults with subjective memory complaints and in those with suspected MCI [94,95], while a 24-week trial of B. breve MCC1274 in patients with suspected MCI attenuated progression of brain atrophy on Magnetic Resonance Imaging (MRI) and improved selected cognitive subscales [96]. Polyphenol-rich dietary patterns also appear to exert neuroprotective effects. In the PREDIMED randomized trial, a Mediterranean diet supplemented with extra-virgin olive oil or nuts resulted in better global cognitive performance over approximately four years compared with a low-fat control diet [97]. Targeted polyphenol interventions, such as high-dose resveratrol in mild-to-moderate AD, have demonstrated changes in AD-related cerebrospinal fluid biomarkers and brain volume loss, albeit with limited effects on global cognition [98]. Similarly, daily intake or supplementation with green tea catechins or matcha has been associated with modest improvements in specific cognitive domains in middle-aged and older adults with subjective cognitive decline or MCI [99,100]. Overall, recent meta-analyses on probiotic supplementation in cognitive impairment report inconclusive results: while some suggest modest cognitive benefits, others, particularly in AD and MCI populations, have not demonstrated significant effects on global cognitive outcomes [101,102]. However, it has to be highlighted that most available studies are cross-sectional and methodologically heterogeneous, preventing causal inference and limiting reproducibility across sampling and sequencing approaches. Mechanistic data rely largely on animal models that do not fully reflect human oral–neural physiology, while clinical interventions remain underpowered, short in duration, and focused on surrogate outcomes.

## 5. Future Perspectives in Oral Microbiota Research

The study of the oral microbiota has undergone a profound transformation in recent years, moving beyond the scope of traditional oral diseases to encompass its systemic impacts, including its emerging role in neurodegenerative disorders such as AD. Despite these advances, several key areas remain underexplored and warrant focused investigation in the coming years.

First, there is a critical need for longitudinal, large-scale cohort studies to better understand the temporal dynamics of oral microbiota composition across the lifespan and in response to external influences such as aging, diet, medication, and systemic disease. Most current studies are cross-sectional, limiting insights into causal relationships between oral dysbiosis and neurodegenerative processes. Establishing whether microbial alterations precede or follow cognitive decline will be essential in determining their etiological relevance to AD. Second, the development and application of multi-omics approaches (including metagenomics, metatranscriptomics, proteomics, and metabolomics) offer unprecedented opportunities to elucidate the functional attributes of the oral microbiome. Functional profiling could reveal microbial metabolic pathways and virulence factors involved in systemic inflammation, immune modulation, and neural signaling, thereby identifying potential biomarkers or therapeutic targets. Integration of oral microbiome data with host genetics, immunological parameters, and neuroimaging findings could help build predictive models of disease susceptibility and progression. Another promising direction lies in interventional studies that assess the effectiveness of microbiota-modulating strategies, such as probiotics, prebiotics, dietary interventions, and oral hygiene optimization, in preventing or mitigating cognitive decline. While preliminary evidence supports the impact of these interventions on oral and systemic health, their ability to influence the oral–brain axis remains largely untested in human clinical trials. Particular attention should be paid to the selection of microbial strains or compounds with well-defined mechanisms of action, as well as the duration, dosage, and delivery method of interventions. Moreover, the neural routes of microbial translocation, such as the trigeminal pathway, require deeper exploration to clarify their roles in CNS invasion and inflammation. Understanding how microbial components bypass or compromise the blood–brain barrier will be vital for designing strategies to block or mitigate these processes. Finally, the oral microbiome should be integrated into a broader systems biology framework that accounts for its interactions with the gut microbiome, immune system, and endocrine pathways. The oral and gut microbiota are not isolated compartments but communicate via immunological and metabolic signaling, forming a complex bidirectional network that can influence brain health. Future research must consider these interconnected ecosystems to fully capture the pathophysiological landscape of neurodegenerative diseases. As analytical tools, computational modeling, and artificial intelligence continue to evolve, they will enhance our capacity to interpret complex microbiome datasets and uncover novel associations. Ultimately, advancing our understanding of the oral microbiota’s role in systemic and neurological health holds great promise for developing personalized, non-invasive, and preventive strategies against conditions such as Alzheimer’s disease.

## 6. Conclusions

The present review highlights the emerging role of the oral microbiota as a potentially important contributor to brain aging and neurodegenerative processes. Beyond its established functions in oral and systemic health, the oral microbiome may influence cognitive decline through interconnected inflammatory, vascular, and neuroimmune mechanisms operating along the oral–brain axis. In this context, chronic oral dysbiosis, most prominently associated with periodontal disease, can promote sustained systemic inflammation, facilitate microbial translocation, and compromise blood–brain barrier integrity, thereby creating a permissive environment for neuroinflammation and neurodegeneration. These processes converge on key pathological features of Alzheimer’s disease, including microglial activation, amyloid-β accumulation, and tau pathology. Collectively, this perspective extends the traditional neuron-centric view of neurodegeneration by highlighting the contribution of peripheral biological systems and chronic low-grade inflammation as central drivers of disease progression.

Building on these insights, the oral microbiota is an integral part of human health, functioning not only as a major determinant of oral homeostasis but also as a mediator of systemic processes that extend to the CNS. Increasing evidence supports a multifaceted relationship between oral microbial dysbiosis and Alzheimer’s disease, potentially mediated through inflammatory pathways, microbial translocation via the bloodstream and neural circuits, and disruption of the blood–brain barrier. Diet emerges as a key modifiable factor shaping the oral microbial community and, by extension, influencing neuroinflammation and cognitive outcomes. Functional foods, probiotics, prebiotics, and bioactive compounds such as (poly)phenols offer promising avenues to modulate the oral microbiota and restore microbial eubiosis, with potential downstream benefits for brain health.

However, current evidence remains largely observational and mechanistic, and important gaps persist. Most available studies are cross-sectional or derived from experimental models, limiting causal inference and translational applicability. Future research should prioritize longitudinal human studies, standardized microbiome and cognitive assessments, and well-designed clinical trials targeting the oral microbiota to determine whether modulation of this ecosystem can meaningfully alter the trajectory of cognitive decline.

In summary, the interplay among the oral microbiota, diet, and brain health represents a rapidly evolving field with significant implications for the early detection and prevention of neurodegenerative diseases. As our understanding deepens, the oral cavity may emerge not only as an indicator of systemic health but also as a targetable interface for interventions aimed at preserving cognitive function and promoting healthy aging.

## Data Availability

No new data were created or analyzed in this study. Data sharing is not applicable to this article.

## Abbreviations

The following abbreviations are used in this manuscript:

Aβ	β-amyloid
AD	Alzheimer’s disease
BBB	Blood–brain barrier
CNS	Central nervous system
EPS	Extracellular polysaccharide
FOS	Fructo-oligosaccharides
GCF	Gingival crevicular fluid
GOS	Galacto-oligosaccharides
IL-1α	Interleukin-1α
IL-1β	Interleukin-1β
IL-6	Interleukin-6
MCI	Mild cognitive impairment
MMPs	Matrix metalloproteinases
MMSE	Mini-mental state examination
MRI	Magnetic resonance imaging
NO	Nitric oxide
PD	Periodontal disease
ROS	Reactive oxygen species
TNF-α	Tumor necrosis factor-α
